# Retinal layer thickness as a potential marker for future cardiovascular events - a UK Biobank cohort study using optical coherence tomography

**DOI:** 10.1007/s00415-026-14175-4

**Published:** 2026-10-15

**Authors:** Martin Hänsel, Julian Deseoe, Lisa Herzog, Neda Davoudi, Veronika Kana, Björn Menze, Beate Sick, Susanne Wegener

**Affiliations:** 1 Department of Neurology and Clinical Neuroscience Center, University Hospital Zurich and University of Zurich, Frauenklinikstrasse 26, 8091 Zurich, Switzerland; 2 Department of Information Technology and Electrical Engineering, ETH Zurich, Institute for Biomedical Engineering, Zurich, Switzerland; 3 Department of Quantitative Biomedicine, University of Zurich, Zurich, Switzerland; 4 Department of Neurology, University Hospital Zurich and University of Zurich, Zurich, Switzerland; 5 Epidemiology, Biostatistics and Prevention Institute, University of Zürich, Zurich, Switzerland

**Keywords:** Stroke, Myocardial infarction, Cerebrovascular disease, Optical coherence tomography, Retinal nerve fiber layer, Ganglion cell-inner plexiform layer

## Abstract

**Aims:**

Cardiovascular disease (CVD) is the leading cause of death worldwide. For CVD prevention, effective screening is needed to identify high-risk individuals. Optical coherence tomography (OCT) provides a noninvasive, low-cost “quick look” into the brain and has recently shown thinning of certain retinal layers in patients with previous CVD. We explored if OCT-derived retinal layer thicknesses could predict future major adverse cardiovascular events (MACE: stroke/myocardial infarction/cardiovascular death).

**Methods:**

In this retrospective cohort study, data of 51,293 participants from the UK Biobank were analyzed. We used cause-specific hazard models to evaluate the association of retinal nerve fiber layer thickness (RNFL) and ganglion cell-inner plexiform layer (GCIPL) thickness with incident MACE, adjusting models for cardiovascular risk factors (CVRF) and performed subanalyses for association with incident stroke and myocardial infarction.

**Results:**

Median follow-up time was 14.7 years. Both RNFL thickness (aHR 0.92, 95% CI 0.89–0.96, p < 0.001) and GCIPL thickness (aHR 0.96, 95% CI 0.92–1.00, *p* = 0.04) were statistically associated with incident MACE in cause-specific hazard models adjusted for CVRF. Interestingly, RNFL thickness was more strongly associated with incident myocardial infarction (aHR 0.94, 95% CI 0.88–0.99, *p* = 0.02) while GCIPL was more strongly associated with incident stroke (aHR 0.90, 95% CI 0.83–0.96, *p* = 0.003).

**Conclusion:**

RNFL and GCIPL thicknesses are associated with future MACE, even after adjusting for CVRF. OCT-measured retinal layer thickness could contribute to the assessment of cardiovascular risk.

**Graphical abstract:**

**Key question**:

Can measurement of retinal layer thickness by optical coherence tomography (OCT) improve prediction of incident cardiovascular events?

**Key finding**:

Retinal nerve fiber layer (RNFL) thickness and ganglion cell-inner plexiform layer (GCIPL) thickness are associated with incident major adverse cardiovascular events (MACE). They lead to modest improvements in prediction of MACE when both layers were added to a cause-specific hazard model based on age and sex.

**Take-home message**:

Measures of retinal layer thickness using OCT have the potential to improve cardiovascular risk screening.

**Figure:**
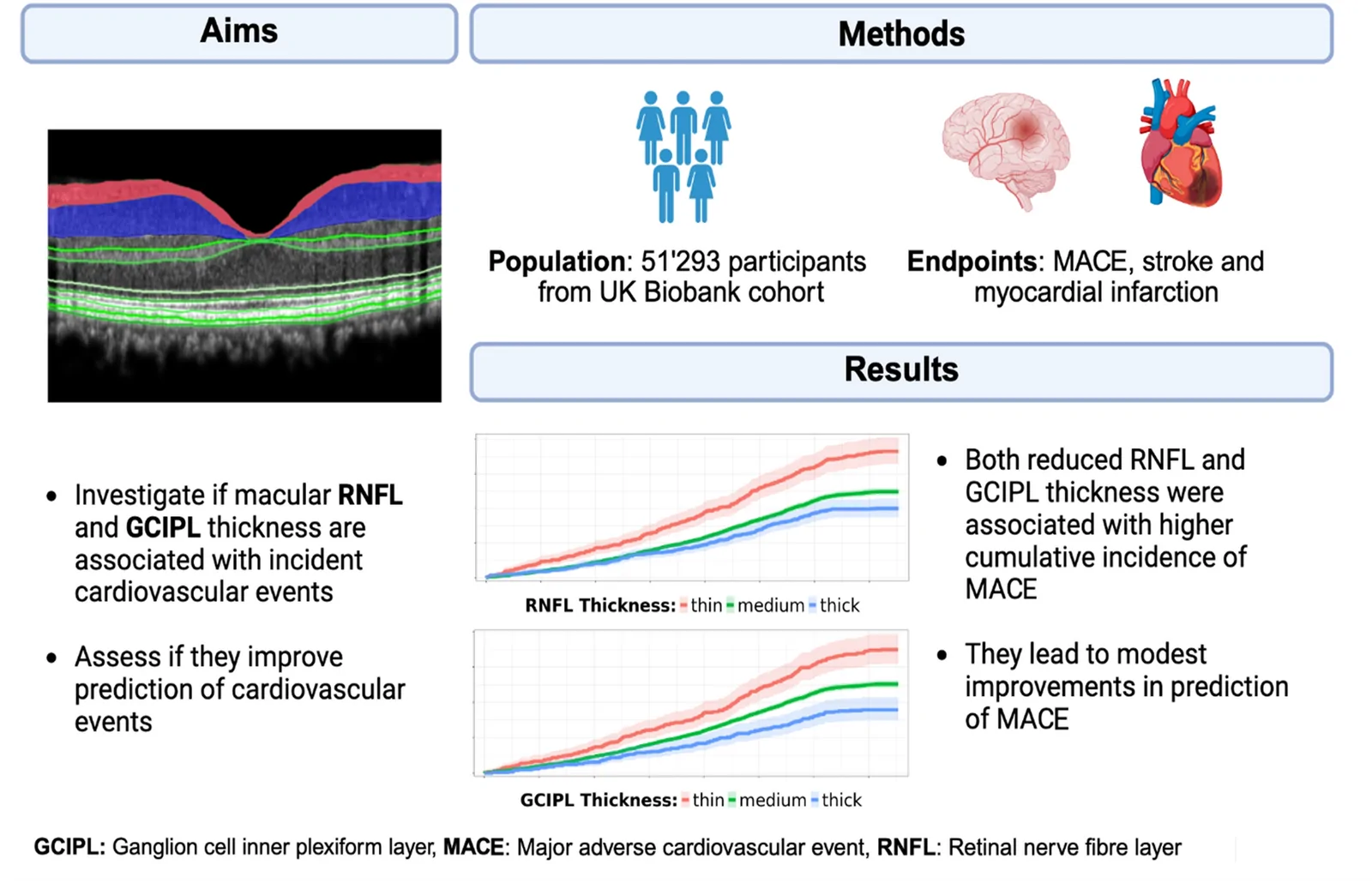

**Supplementary Information:**

The online version contains supplementary material available at https://doi.org/10.1007/s00415-026-14175-4.

## Lay summary

**Table:** 

This study aimed at identifying predictors of major adverse cardiovascular events (MACE) by analyzing the thickness of retinal layers in patients with and without cardiovascular disease (CVD) using optical coherence tomography (OCT).
Both retinal nerve fiber layer (RNFL) thickness (HR 0.92, 95% CI 0.89–0.96, *p* < 0.001) and ganglion cell-inner plexiform layer (GCIPL) thickness (HR 0.96, 95% CI 0.92–1.00, *p* = 0.04) were significantly associated with incident MACE (adjusted for traditional cardiovascular risk factors) during a median follow-up time of 14.7 years.
RNFL and GCIPL thickness are associated with future MACE, emphasizing their potential for screening individuals at risk of future CVD.

## Introduction

Despite advances in prevention and treatment, cardiovascular diseases (CVD) remain the leading cause of death worldwide [1]. To prevent major adverse cardiovascular events (MACE: stroke, myocardial infarction and cardiovascular death) effectively, it is important to identify patients at risk. In clinical practice, risk stratification for MACE is primarily achieved by calculating risk scores incorporating cardiovascular risk factors (CVRF) in combination with additional diagnostic measures [2–10]. However, better predictors are needed. In recent years, the decrease in retinal layer thickness, as measured by optical coherence tomography (OCT), has emerged as a potential marker for CVD [11–16]. OCT uses light waves to generate detailed cross-sectional images of the ten retinal layers and provides a noninvasive, side-effect-free, “quick look” into the brain [17]. Several studies have investigated the relationship between OCT parameters and previous MACE. These have shown that patients with previous MACE show thinning of the ganglion cell-inner plexiform layer (GCIPL) and the retinal nerve fiber layer (RNFL) [11–14, 16]. The pathophysiological relationship between reduced RNFL/GCIPL thickness and CVD remains largely unclear. In the context of CVD and CVRF like arterial hypertension, a thinning of the RNFL has been explained by disturbances in the retinal microcirculation leading to microvascular ischemia [12, 18–20].

While data on the association of GCIPL and RNFL thickness with *previous* MACE exists, it is rarely investigated if GCIPL and/or RNFL thickness could indicate *incident* cardiovascular events. Using a large, retrospective population-based cohort from the UK Biobank, we aimed at evaluating the use of OCT-derived measures of retinal layer thicknesses for risk stratification of incident MACE. In subanalyses, we investigated risk stratification for incident stroke and myocardial infarction. As secondary outcome, we tested different prediction models including the RNFL/GCIPL and different CVRF, also including survival analysis, calculating for the competing event. Further, we generate normative values for the RNFL and GCIPL.

## Methods

### Study design, participants, and setting

We used data from the UK Biobank (UKBB), a large population-based multicenter cohort study with over 500,000 participants [21]. Adults, aged 40–69 years and residing in the United Kingdom, were recruited between 2006 and 2010. As part of the assessments, a portion of UKBB participants underwent OCT imaging during their initial visit (2006–2010). Data were analyzed retrospectively in 2025. All participants provided written informed consent. This analysis was performed under the UK Biobank application ID 467527, approved on the 13th December 2024. We worked with the release of the UKBB data from 19th March 2025. The investigation is in line with the principles set out in the Declaration of Helsinki.

### Baseline characteristics

We divided the participants into two groups based on patient characteristics: those with and those without prior cardiovascular events (ischemic/hemorrhagic stroke or myocardial infarction), using the algorithmically defined outcomes in the UKBB. For each of the participants, we ascertained the CVRF shown in Table 1. Age was the age at the initial visit. Sex was also recorded at the initial visit. The smoking status was taken from self-report and classified as current, previous or never. BMI was also recorded at the initial visit in the UKBB data. Hypertension, diabetes, dyslipidemia, and atrial fibrillation were identified through linked health records using the ICD-10 codes shown in Supplementary Table 1. Patients with incomplete data on CVRF were excluded. For investigation of RNFL and GCIPL associations with incident MACE, we excluded all participants with previous stroke or myocardial infarction.

**Table 1:** Patient characteristics and retinal layer thicknesses of the included participants with and without prior cardiovascular events

	No prior MACE	Prior MACE	*p* value
*n*	49,787	1506	
Age (mean, SD)	56.69 (8.14)	61.48 (6.69)	< 0.001
Sex (women, %)	27,820 (55.9)	456 (30.3)	< 0.001
Hypertension (%)	11,888 (23.9)	887 (58.9)	< 0.001
Diabetes (%)	1,823 (3.7)	188 (12.5)	< 0.001
Dyslipidemia (%)	6,714 (13.5)	820 (54.4)	< 0.001
BMI (SD)	27.21 (4.71)	28.72 (4.85)	< 0.001
*Smoking* (%)			< 0.001
Never	27,998 (56.2)	582 (38.6)	
Current	4,805 (9.7)	190 (12.6)	
Previous	16,984 (34.1)	734 (48.7)	
Atrial fibrillation (%)	632 (1.3)	152 (10.1)	< 0.001
*Retinal layers* (*mean SD, µm*)			
RNFL	29.70 (6.41)	28.97 (6.02)	< 0.001
GCIPL	74.21 (8.75)	72.99 (8.54)	< 0.001
INL	32.22 (3.88)	32.33 (3.73)	0.265
ELM.ISOS	23.37 (2.56)	23.29 (2.11)	0.257
ISOS.RPE	37.55 (5.50)	37.25 (4.95)	0.036

*p* values were calculated using unpaired T tests for continuous variables and Chi-squared tests for categorical variables

*BMI* body mass index, *MACE* major adverse cardiovascular event (stroke/myocardial infarction), *RNFL* retinal nerve fiber layer, *GCIPL* ganglion cell-inner plexiform layer, *INL* inner nucleus layer, *ELM* external limiting membrane, *RPE* retinal pigment epithelium, *ISOS* inner segment/outer segment junction

### Retinal imaging

**Fig. 1:**
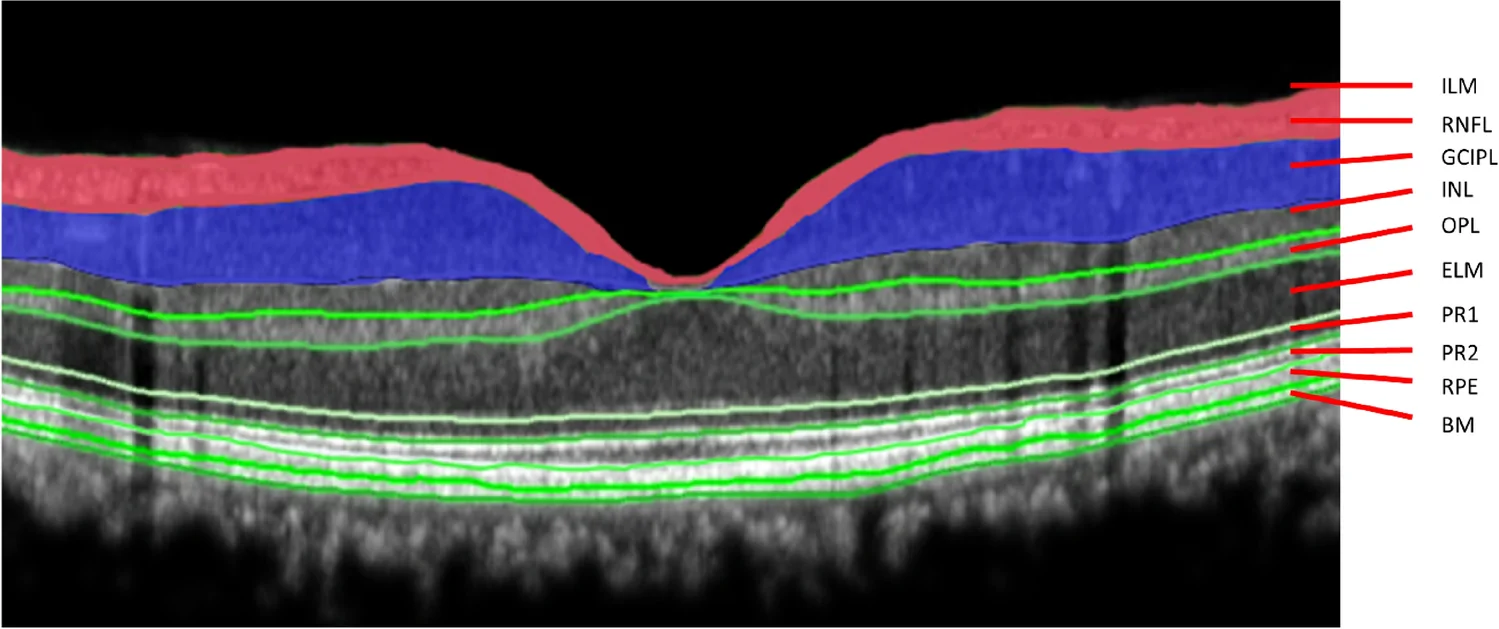
Automated segmentation of an optical coherence tomography (OCT) image set. The figure shows the nine intraretinal boundaries, which were derived by automated segmentation with Heidelberg Eye Explorer 2.6.4. Red: RNFL, Blue: GCIPL. *ILM* internal limiting membrane, *RNFL* retinal nerve fiber layer, *GCIPL* ganglion cell layer, *INL* inner nuclear layer, *OPL* outer plexiform layer, *ELM* external limiting membrane, *PR1* photoreceptor layer 1, *PR2* photoreceptor layer 2, *RPE* retinal pigment epithelium, *BM* Bruch’s membrane

The OCTs were done with a raster scan protocol, 6 mm × 6 mm in the area of the macula, and consisting of 128 B-scans with the “spectral domain” OCT device (3D OCT-1000 Mark II, Topcon, Japan) by trained UKBB technicians [22]. The Topcon Advanced Biomedical Imaging Laboratory developed custom image segmentation software for rapid, automated analysis of retinal thickness with quality scores [22]. We worked with data from the first UK Biobank assessment. The right eye was chosen for analysis. If that was unavailable, the left eye was chosen instead [11, 22]. Participants were excluded if neither eye was available or if they reported having an eye disease. According to previous studies using OCT, we excluded the poorest 20% of images based on quality scores [23]. The following retinal layers were analyzed (Fig. 1): (1) RNFL thickness, (2) GCIPL thickness, (3) inner nuclear layer (INL) thickness, (4) distance measured between the INL and the external limiting membrane (INL.ELM), (5) distance measured between the external limiting membrane (ELM) and the inner and outer photoreceptor segments (ELM.ISOS), and (6) distance measured between the inner and outer photoreceptor segments (ISOS) and the retinal pigment epithelium (ISOS.RPE).

### Statistical analysis

In a first step, the descriptive data of the patients’ characteristics and the different retinal layers were analyzed using mean values, including the standard deviation (SD), as well as the absolute numbers (%). To investigate if our exclusion of participants with insufficient OCT quality and missing covariates led to biases, we compared cohort characteristics before and after exclusion of these participants. Participants were stratified by prior CVD (stroke or myocardial infarction), and significant differences between the two groups were tested using a two-sided, unpaired *t* test. The results were considered significant if *p* was < 0.05. Retinal layer thicknesses were stratified by CVD prior to the OCT measurement and visualized by boxplots. Due to the significant differences in the findings of the RNFL and GCIPL and based on previous literature, further subanalyses were performed only on these two layers. The correlation of RNFL and GCIPL per eye was visualized by pair plots. Boxplots and scatterplots were used to visualize RNFL and GCIPL depending on different CVRF.

For investigation of RNFL and GCIPL thickness with incident MACE in a cohort without previous CVD, we performed a competing risk analysis with MACE (comprising stroke, myocardial infarction, or cardiovascular death) as primary endpoint and non-cardiovascular death as competing risk. The date of the first assessment was used as the start date. Participants without MACE or non-cardiovascular death until 1st January 2025 were censored. We calculated both cause-specific and subdistribution hazard models according to recommendations from the literature [24]. Separate models for associations of RNFL and GCIPL thickness were built. Both unadjusted models and models adjusted for CVRF shown in Table 1 were calculated. Details on ascertainment of CVRF can be found in Table S1. We screened for sex-specific effects by including interaction terms between sex and RNFL/GCIPL thickness. We also screened for nonlinear associations of MACE risk with GCIPL and RNFL thickness, respectively, using restricted cubic spline with four nodes. Significance of nonlinear components was assessed using partial F test. We used likelihood ratio tests to determine if adding RNFL/GCIPL layer thicknesses added significant information to the models. In a subanalysis, we tested the association of RNFL and GCIPL with incident stroke and myocardial infarction separately, treating all cause death as competing event. For all survival models, we assessed Schoenfeld residuals to test the proportional hazards assumption. To complete the survival analysis, we also calculated the same models for the competing event. We report hazard ratios (HR) with its 95% confidence intervals (CI). We standardized RNFL and GCIPL thickness to have a mean of 0 and a standard deviation of 1 to improve comparability of effect sizes. We then classified the participants into three groups (thin, medium and thick RNFL/GCIPL) based on the mean ± standard deviation cut-offs for RNFL and GCIPL thickness. Cumulative incidence of MACE at 10 years was calculated for each of the groups, and cumulative incidence function curves were plotted.

Further, we compared the area under the receiver operating characteristic curve (ROC AUC) and Brier Scores of cause-specific and subdistribution hazard models based on age and sex and models based on age, sex, RNFL and GCIPL for predicting incident MACE at 10 years using fivefold cross-validation. We decided to compare to a model based on age and sex as this is the generic information available to clinicians before screening for cardiovascular risk factors. We also compare the same metrics when adding GCIPL and RNFL thickness to fully adjusted models. Finally, we calculated age- and sex-specific mean and standard deviations for RNFL and GCIPL thickness. All analyses were performed using the R programming language version 4.4.0 on the UK Biobank research analysis platform [25]. The different R programming packages for implementation, along with descriptions of advanced statistical methods, can be found in the Supplementary material.

## Results

### Patient characteristics and thickness of retinal layers depending on prior CVD

**Fig. 2:**
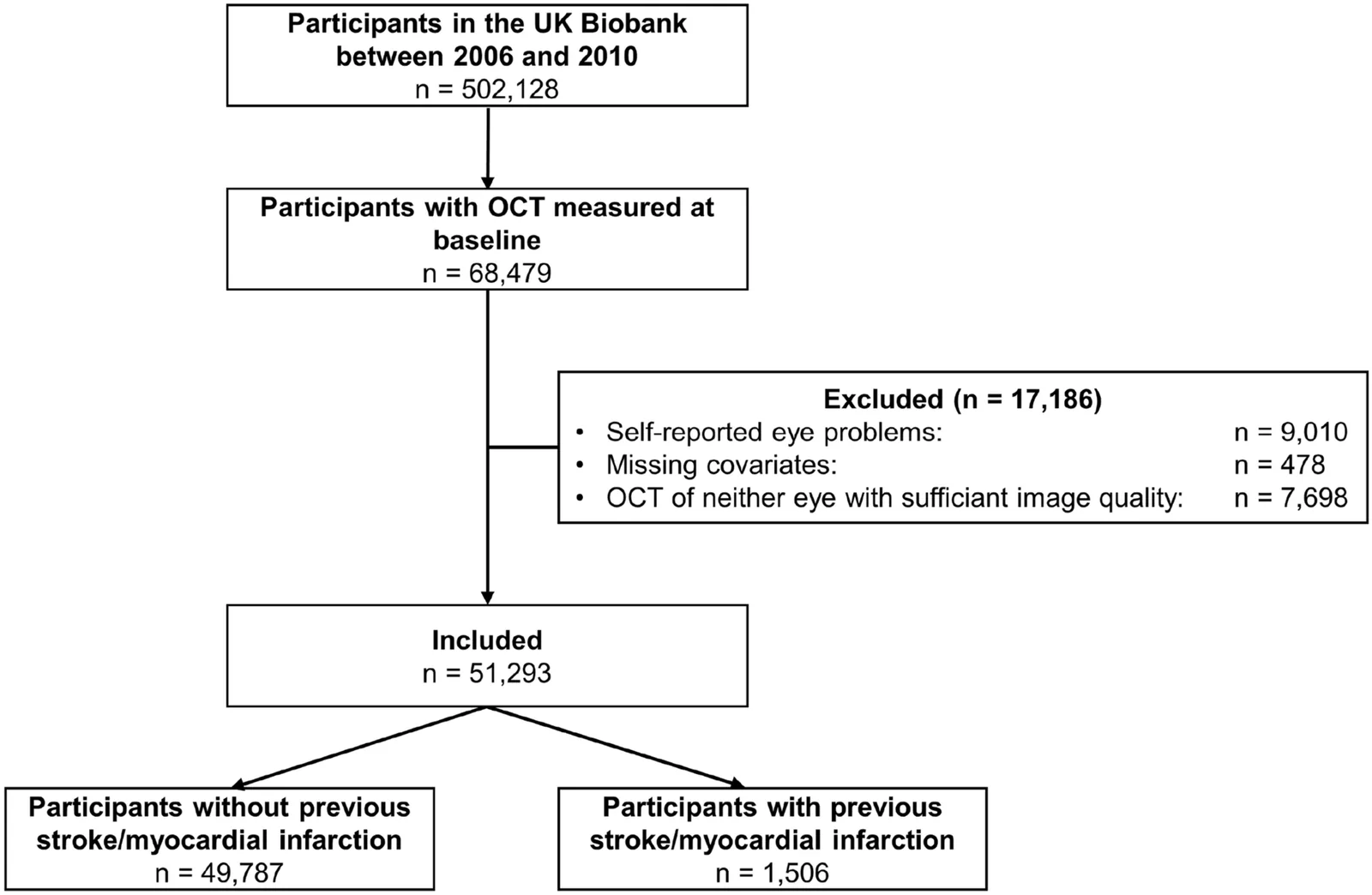
Flowchart of the inclusion and exclusion criteria for UK Biobank participants between 2006 and 2010

Of all 502,128 UKBB participants, 51,293 participants (55.1% women) were included in our analysis (Fig. 2). Of those participants, 1506 (2.9%) had prior CVD (stroke or myocardial infarction). Those with CVD were older, more likely male and had a higher BMI compared to those without cardiovascular events (Table 1, all *p* < 0.001). They also had more CVRF like hypertension, diabetes, hyperlipidemia, atrial fibrillation, and were more often current or previous smokers (all p < 0.001). Cohort characteristics before and after removal of participants with low-quality OCT data and missing covariates were highly similar, meaning there is no evidence that bias was introduced through this participant exclusion (Table S2).

**Fig. 3:**
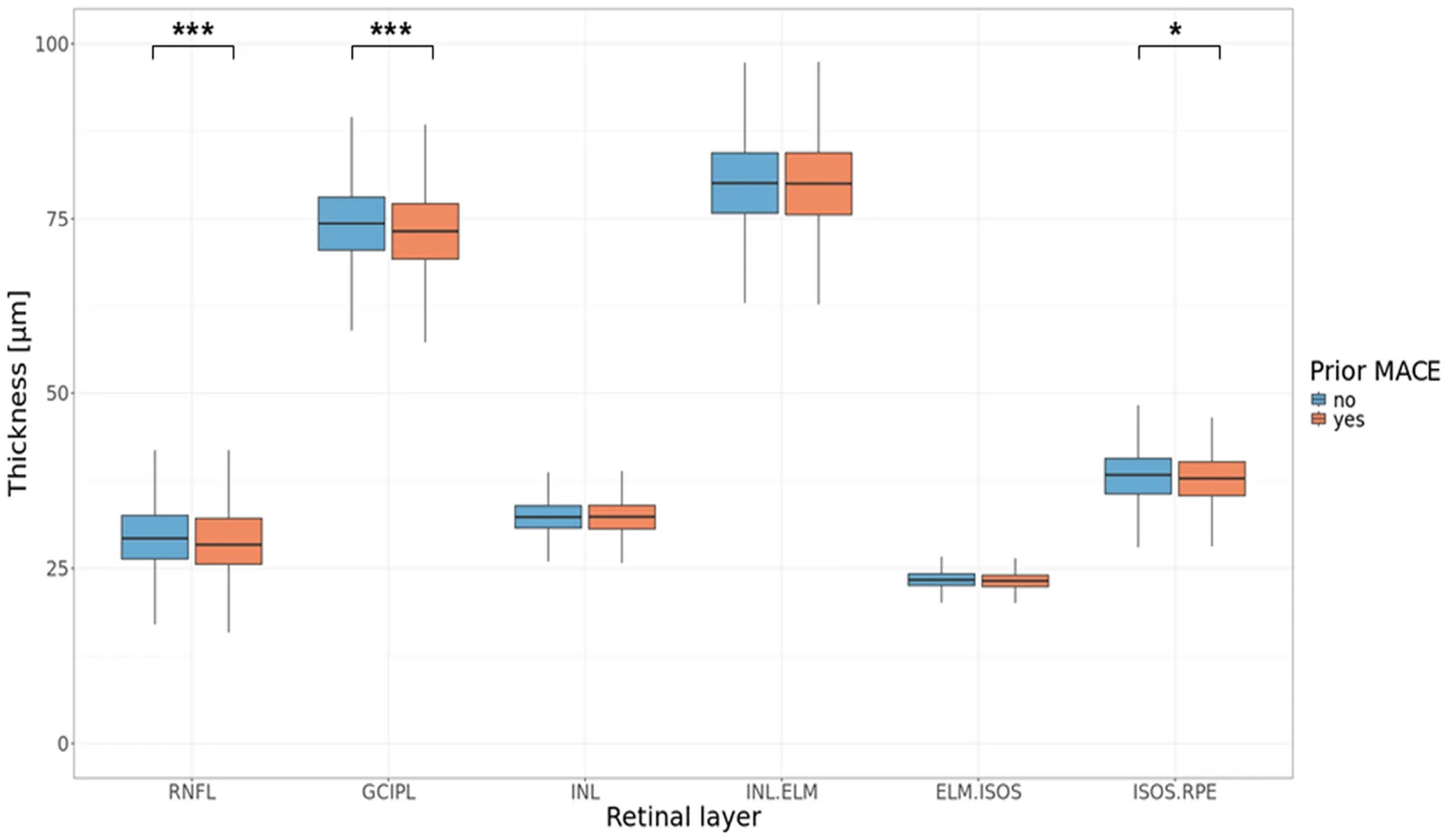
Retinal layer thickness stratified by cardiovascular event prior to first OCT (*n* = 51,293). Boxplots show median, interquartile range, and minimum and maximum values. Outliers defined as values more than two standard deviations above or below the mean are not shown. *RNFL* retinal nerve fiber layer, *GCIPL* ganglion cell-inner plexiform layer, *INL* inner nucleus layer, *ELM* external limiting membrane, *RPE* retinal pigment epithelium, *ISOS* inner segment/outer segment junction. *MACE* major adverse cardiovascular events. *p < 0.05. ***p < 0.001. *p* values calculated by unpaired T test

**Fig. 4:**
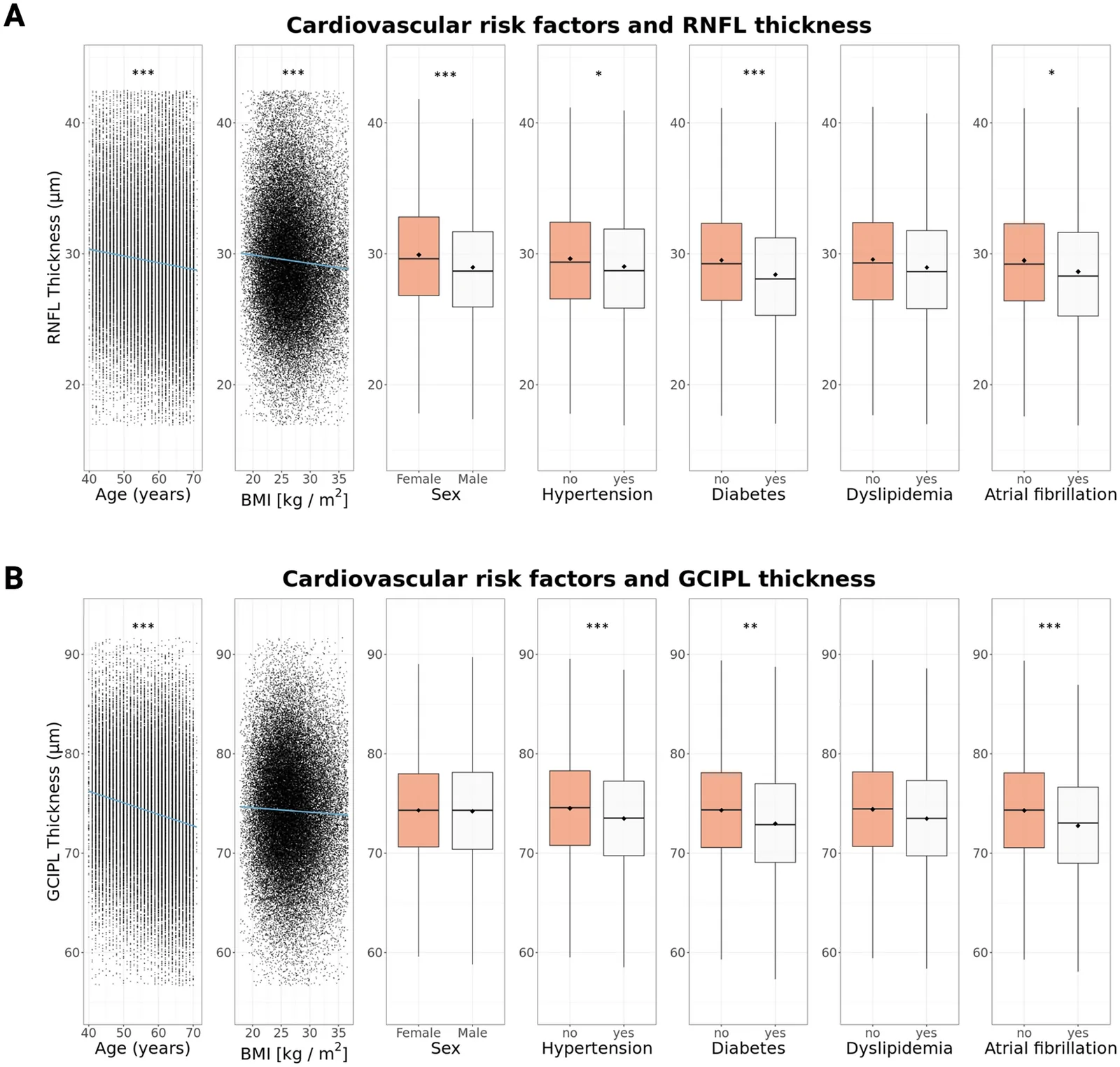
Association of RNFL and GCIPL thickness with cardiovascular risk factors. **A** shows RNFL thickness depending on cardiovascular risk factors (CVRF), age, and sex. **B** shows GCIPL thickness depending on CVRF, age, and sex. Regression lines calculated by simple linear regression. Boxplots show median, interquartile range, and minimum and maximum values. Stars indicate statistically significant associations in multivariate linear regression models including all shown parameters (Table S3-S4: *p < 0.05, **p < 0.01, ***p < 0.001). N = 51,293, outliers further from the mean than two standard deviations are not shown)

When comparing the retinal layers of participants with and without prior CVD, three layers were significantly thinner after a stroke or myocardial infarction: (1) RNFL (28.97 µm ±6.02 vs. 29.70 µm ±6.41, p < 0.001), (2) GCIPL (72.99 µm ±8.54 vs. 74.21 µm ±8.75, p < 0.001), and (3) ISOS.RPE (37.25 µm ±4.95 vs. 37.55 µm ±5.50, *p* = 0.036) (Fig. 3). Thickness of the other three layers (INL, INL.ELM, and ELM.ISOS) did not differ significantly between groups (Table 1/Fig. 3). Based on these results and previous literature findings, we focused on the RNFL and the GCIPL for further analyses. Figure 4 and Tables S3–S4 show the associations of the RNFL and GCIPL thickness with CVRF, age, and sex. While both the RNFL and the GCIPL were strongly associated with age (*p* < 0.001), the GCIPL was more strongly associated with hypertension and atrial fibrillation (*p* < 0.001). The RNFL was thicker in subjects with female sex, lower BMI, no smoking history, and no diabetes (*p* < 0.001).

Further, we report age- and sex-stratified mean and standard deviations of GCIPL and RNFL thicknesses (Tables S5–S6). These also show a decrease in GCIPL and RNFL thickness with increasing age and men having thinner GCIPL/RNFL on average.

### RNFL and GCIPL thickness are associated with incident MACE

**Fig. 5:**
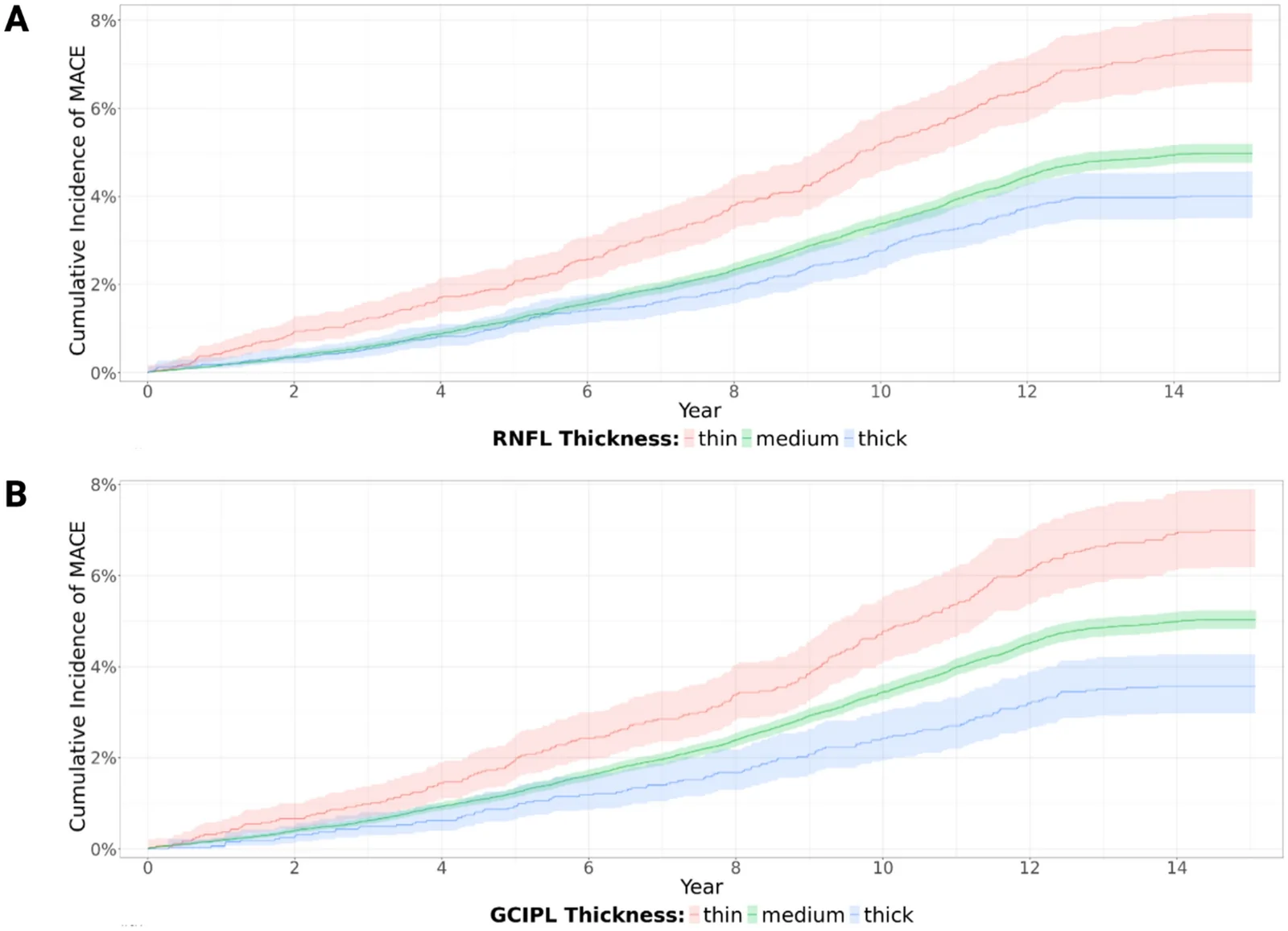
Timeline of cumulative incidence of major adverse cardiovascular events depending on RNFL and GCIPL thickness. Lines show cumulative incidence function for MACE; shaded area indicated 95% confidence intervals. In the first graph (**A**), participants are grouped by RNFL thickness, in the second graph (**B**), participants are grouped by GCIPL thickness. N = 49,787

In a second step, we analyzed the associations of RNFL/GCIPL thickness with incident MACE with a median follow-up time of 14.7 years (min: 0.0/ max: 15.1). Using unadjusted cause-specific hazard models, we found a significantly inverse association between RNFL/GCIPL and risk of future stroke, myocardial infarction, and MACE (*p* < 0.001 for all). Detailed results of the unadjusted cause-specific hazard models are provided in the supplementary material (Supplementary Table S7). Participants with a thin RNFL were almost twice as likely to suffer MACE (5.19%, 95% CI 4.57–5.90%) in the following 10 years compared to participants with a thick RNFL thickness (2.78%, 95% CI 2.37–3.25%) (Fig. 5). Similarly, we found that the cumulative incidence of MACE in participants with a thick GCIPL was almost half (2.42%, 95% CI 1.95–3.02%) after 10 years, compared to participants with a thin GCIPL (4.76%, 95% CI 4.10–5.53%). Hazard ratios for MACE remained significant but decreased in size when adjusting models for CVRF (for RNFL: HR 0.92, 95% CI 0.89–0.96, *p* < 0.001; for GCIPL: HR 0.96, 95% CI 0.92–1.00, *p* = 0.04) (Table 2). Regression coefficients for all covariates can be found in Tables S8 and S9. No significant interactions were found between sex and RNFL thickness (*p* = 0.47) or sex and GCIPL thickness (*p* = 0.88). No significant nonlinear associations were found for the associations of GCIPL (*p* = 0.27) and RNFL (*p* = 0.37) with MACE risk. Notably, subanalyses of MACE revealed slight differences between GCIPL and RNFL concerning associations with stroke and myocardial infarction. For stroke alone, the fully adjusted models for CVRF showed a significant association between GCIPL (HR: 0.90 95% CI 0.83–0.96, *p* = 0.003), and a weaker, non-significant association with RNFL thickness (HR: 0.95 95% CI 0.88–1.01, *p* = 0.08). Regarding myocardial infarction alone, a significant association was seen with RNFL (HR: 0.95, 95% CI 0.88–0.99. *p* = 0.02), while a weaker, non-significant association was found with GCIPL (HR: 0.98, 95% CI 0.92–1.03, *p* = 0.45). Similar differences were also found in unadjusted models (Supplementary Table S7). Subdistribution hazard models and models for the competing event (non-cardiovascular death and death) were calculated (Supplementary Tables S10–S15). ROC AUC and Brier Scores for prediction of incident cardiovascular events at 10 years showed modest improvement when RNFL thickness and GCIPL thickness were added to a cause-specific hazard model based on age and sex (ROC AUC: 0.708 95% CI 0.7–0.717 vs. 0.710 95% CI 0.701–0.718, *p* = 0.04) (Table 3). Differences were smaller in fully adjusted models. Subdistribution hazard models showed similar results (Table S16).

**Table 2:** Cause-specific hazard models fully adjusted for cardiovascular risk factors

Layer	Hazard ratio	95% CI lower	95% CI upper	*P* value	Likelihood ratio test
*MACE*					
GCIPL	0.96	0.92	1.00	0.04	0.03
RNFL	0.92	0.89	0.96	< 0.001	< 0.001
Events: 2526		Median FU: 14.6 years			
*Stroke*					
GCIPL	0.90	0.83	0.96	0.003	0.003
RNFL	0.94	0.88	1.01	0.08	0.08
Events: 882		Median FU: 14.7 years			
*Myocardial infarction*					
GCIPL	0.98	0.92	1.03	0.44	0.45
RNFL	0.94	0.88	0.99	0.02	0.02
Events: 1411		Median FU: 14.7 years			

Hazard ratios with 95% confidence intervals (CI) are shown. Column *p* value shows P values of the hazard ratio from the fully adjusted cause-specific hazard model. Column *Likelihood ratio test* shows p values when comparing models with and without retinal layer thickness with the likelihood ratio test

*GCIPL* ganglion cell-inner plexiform layer, *RNFL* retinal nerve fiber layer, *MACE* major adverse cardiovascular event. *N* = 49,787

**Table 3:** Predictive performance of models for predicting 10-year risk of major adverse cardiovascular events

Model	AUC	95% CI	Brier (%)	95% CI	*P* value
Age + sex	0.7082	0.6998 - 0.7167	3.283	3.139 - 3.427	
Age + sex + RNFL + GCIPL	0.7095	0.7011 - 0.7180	3.282	3.138 - 3.426	0.041
Age + sex +CVRF	0.7400	0.7321 - 0.7481	3.257	3.115 3.399	
Age + sex + CVRF + RNFL + GCIPL	0.7402	0.7322 - 0.7482	3.256	3.114 - 3.398	0.74

ROC AUC values for predicting 10-year risk of major adverse cardiovascular events for cause-specific hazard model based only on age and sex and models with GCIPL and RNFL thickness added as well as analogous fully adjusted models. *p* values were calculated using DeLong’s test for comparing ROC Curves

*CVRF* cardiovascular risk factors, *GCIPL* ganglion cell-inner plexiform layer, *RNFL* retinal nerve fiber layer, *ROC AUC* area under the receiver operating curve. *N* = 49,787

## Discussion

Our findings from 49,787 participants of the UKBB cohort over a median follow-up time of 14.6 years show an association between OCT-measured retinal layer thicknesses and incident cardiovascular events. Both GCIPL and RNFL thickness were significantly associated with incident MACE even when adjusting for a wide range of CVRF. Further, including RNFL and GCIPL thickness in multivariate models based on age and sex led to modest improvements in prediction of cardiovascular events for a timeframe of 10 years. To facilitate interpretation of RNFL and GCIPL thicknesses, we provide age- and sex-specific norm values. Currently, this is the largest study investigating the value of retinal layer thickness measurement for predicting MACE, since the publication of Chen et al. [15].

There is a growing body of evidence leveraging OCT for cardiovascular risk stratification; the most closely related to our study is from Chen et al. [15]. They analyzed 23,563 participants from the UKBB regarding retinal structural abnormalities as biomarkers for CVD events and found an independent association between RNFL thinning and incident cardiovascular risk. In comparison to their work, due to larger sample size and longer follow-up, we were able to show that not only the RNFL, but also the GCIPL is associated with MACE in patients without prior cardiovascular events. Indeed, the RNFL thickness was more strongly associated with incident myocardial infarction while GCIPL was associated more strongly with incident stroke. To facilitate implementation in clinical practice, we here added age- and sex-specific normal values [15]. Other studies have used retinal layer thicknesses for MACE risk stratification in patients with acute coronary syndrome and Type 1 diabetes, although these studies had smaller sample sizes due to their more selective cohorts [26, 27]. Further, a recent Mendelian randomization study demonstrated shared causes for changes in retinal layer thickness and CVD [16]. Finally, a recent study suggests OCT measures could also be linked to cognitive impairment, which is also a predictor for MACE [28].

Examining the retinal layers of patients with prior CVD in comparison to those without, the findings largely align with previous studies [11–14, 16]. Both Lian et al. (patients with ischemic stroke had significantly thinner RNFL and GCIPL), Neoh et al. (patients with coronary heart disease had significantly thinner RNFL), and Majithia et al. (patients with myocardial infarction or stroke had significantly thinner RNFL) found significantly thinner retinal layers compared to normal control groups [11, 13, 14]. Patients with prior CVD were more likely to be male and to have significantly more CVRF, such as hypertension, diabetes, a higher BMI, smoking, and atrial fibrillation [11–14].

We postulate that thinning of the RNFL and GCIPL occurs due to subclinical atherosclerosis of the central retinal artery resulting in chronically reduced blood flow and oxygen supply to the retina. Since atherosclerosis is a systemic disease, changes to the central retinal artery reflect pathology in other organs, including the heart or brain [29]. Interestingly, RNFL thickness was more strongly associated with incident myocardial infarction, while GCIPL thickness was more strongly associated with incident stroke in both unadjusted and adjusted models, although confidence intervals overlapped. These findings align with previous studies showing that patients with a history of myocardial infarction exhibit stronger RNFL thinning, while patients with previous stroke showed stronger GCIPL thinning [11, 13]. The reasons for the differences in associations between RNFL and GCIPL with stroke and myocardial infarction remain unclear. However, as stroke and myocardial infarction are associated with somewhat differing cardiovascular risk profiles and as RNFL and GCIPL show differing associations with individual CVRF due to differences in their vascular supply, it is plausible that RNFL and GCIPL thicknesses reflect different cardiovascular risk profiles resulting in vascular retinal degeneration [30, 31]. Both the RNFL and the GCIPL are supplied by branches of the central retinal artery; they form distinct plexuses with different properties. The RNFL is primarily supplied by the radial peripapillary capillary plexus, whose capillaries run parallel to RNFL axons. In contrast, the GCIPL is supplied by the superficial vascular plexus, which exhibits a lobular architecture [31]. Hence, it is plausible that the two plexuses show different susceptibility to different CVRF due to their differing architectures. The different compositions of the RNFL and GCIPL may also lead to differing susceptibility levels of the different layers to CVRF. This could explain differences in associations with stroke and myocardial infarction. However, this hypothesis needs further evaluation.

In fact, searching for thinning RNFL/GCIPL thickness using OCT measurements could be an innovative new and additive approach to predicting MACE. OCT is a painless procedure that is well established in ophthalmology for screening for conditions such as diabetic retinopathy, macular degeneration, and glaucoma, and is comparatively low cost per session (approximately 100 CHF in Switzerland). The normal values of RNFL and GCIPL could be used in healthy patients aged 49–69 without eye diseases both as part of regular ophthalmological examinations as an additional MACE risk prediction tool, as well as for targeted grading of individuals with a family history of CVD.

### Strengths

The large sample size of the UKBB and strictly standardized study protocol across the entire UK, which allow adjustment for a wide range of CVRF, is a strength of our study. Further, the UKBB contains long-term follow-up of the participants.

### Limitations

However, there are also limitations. First, only participants between 40 and 69 years of age were recruited to the UKBB. The risk of CVD/MACE increases with age. Hence, the population with the highest risk of MACE (above the age of 70 years) was not included in our analysis. Therefore, our results may not apply to all age groups. Further, we excluded patients with prior eye diseases, meaning our findings cannot be generalized to this subgroup. Since subanalyses of ischemic vs. hemorrhagic stroke were not performed, we cannot rule out differences in OCT parameter association between these different stroke entities. Finally, due to the observational, cross-sectional nature of this study, we cannot establish causal links.

### Knowledge gaps

As in all large cohort studies, it is important to distinguish between statistical significance and clinical relevance. To address this point, the value of screening in clinical practice should be tested in prospective studies.

### Future directions

While we demonstrate that GCIPL and RNFL thickness allow risk stratification for incident MACE, further research is needed to improve predictive performance, evaluate patients with wider age ranges, and perform prospective validation. In order to improve MACE prediction, retinal layer thicknesses could be combined with other OCT parameters. For example, parameters extracted from OCT angiography such as vessel density could be combined with models containing RNFL and GCIPL thickness to improve risk stratification for cardiovascular events [32]. Further, measuring RNFL and GCIPL thickness longitudinally might reveal individual trajectories that could be powerful predictors of individual CVD progression or response to preventive treatments. Furthermore, due to the different pathophysiological mechanisms of ischemic vs. hemorrhagic stroke, further analyses should focus more on this knowledge gap.

## Conclusion

This study is an important step toward introducing OCT as a tool for cardiovascular risk screening in clinical practice, showing its potential but also highlighting the need for further research. While we show that RNFL and GCIPL thickness allow risk stratification for incident cardiovascular events, improvement in predictive performance over established CVRF was only modest. Combining RNFL and GCIPL thickness with additional features extracted from OCT angiography might further improve predictive performance, justifying clinical use in screening for cardiovascular risk.

## Supplementary Information

Below is the link to the electronic supplementary material.

Supplementary file1 (DOCX 675 KB)

## Data Availability

The data underlying this article were provided by the UK Biobank by permission. Data will be shared on request to the corresponding author with permission of the UK Biobank.

## Abbreviations

aHR: Adjusted hazard ratio
CI: Confidence interval
CVD: Cardiovascular disease
CVRF: Cardiovascular risk factors
ELM: External limiting membrane
GCIPL: Ganglion cell-inner plexiform layer
HR: Hazard ratio
INL: Inner nuclear layer
ISOS: Inner and outer photoreceptor segments
MACE: Major adverse cardiovascular events
OCT: Optical coherence tomography
RNFL: Retinal nerve fiber layer thickness
ROC AUC: Area under the receiver operating characteristic curve
RPE: Retinal pigment epithelium
SD: Standard deviation
UKBB: UK Biobank

## References

[1] Roth GA, Mensah GA, Johnson CO, Addolorato G, Ammirati E, Baddour LM, et al. GBD-NHLBI-JACC Global Burden of Cardiovascular Diseases Writing Group. Global Burden of Cardiovascular Diseases and Risk Factors, 1990–2019: Update From the GBD 2019 Study. J Am Coll Cardiol. 2020;76(25):2982–3021. 10.1016/j.jacc.2020.11.010. Erratum in: J Am Coll Cardiol. 2021;77(15):1958–1959. 10.1016/j.jacc.2021.02.039 PMC7755038 33309175

[2] Assmann G, Schulte H (1988) The Prospective Cardiovascular Münster (PROCAM) study: prevalence of hyperlipidemia in persons with hypertension and/or diabetes mellitus and the relationship to coronary heart disease. Am Heart J 116(6 Pt 2):1713–1724. 10.1016/0002-8703(88)90220-7 3202078

[3] Assmann G, Schulte H, Cullen P, Seedorf U (2007) Assessing risk of myocardial infarction and stroke: new data from the Prospective Cardiovascular Münster (PROCAM) study. Eur J Clin Invest 37(12):925–932. 10.1111/j.1365-2362.2007.01888.x 18036028

[4] Josephson SA, Sidney S, Pham TN, Bernstein AL, Johnston SC (2008) Higher ABCD2 score predicts patients most likely to have true transient ischemic attack. Stroke 39(11):3096–3098. 10.1161/STROKEAHA.108.514562 18688003

[5] Dratva J, Caviezel S, Schaffner E, Zemp E, de Groot E, Schmidt-Trucksäss A et al (2014) Early detection of subjects at risk for vascular remodelling - results from the Swiss population-based study SAPALDIA. Swiss Med Wkly 144:w14052. 10.4414/smw.2014.14052 25356963

[6] SCORE2 working group, ESC Cardiovascular risk collaboration (2021) SCORE2 risk prediction algorithms: new models to estimate 10-year risk of cardiovascular disease in Europe. Eur Heart J 42(25):2439–2454. 10.1093/eurheartj/ehab309 PMC8248998 34120177

[7] Goff DC, Lloyd-Jones DM, Bennett G, Coady S, D’Agostino RB, Gibbons R, American College of Cardiology/American Heart Association Task Force on Practice Guidelines et al (2013) 2013 ACC/AHA guideline on the assessment of cardiovascular risk: a report of the American College of Cardiology/American Heart Association Task Force on Practice Guidelines. Circulation 129(25 Suppl 2):S49-73. 10.1161/01.cir.0000437741.48606.98 24222018

[8] Razavi AC, Whelton SP, Blumenthal RS, Blaha MJ, Dzaye O (2025) Beyond the Agatston calcium score: role of calcium density and other calcified plaque markers for cardiovascular disease prediction. Curr Opin Cardiol 40(1):56–62. 10.1097/HCO.0000000000001185 PMC11620923 39445716

[9] Polak JF, Pencina MJ, Pencina KM, O’Donnell CJ, Wolf PA, D’Agostino RB Sr. (2011) Carotid-wall intima-media thickness and cardiovascular events. N Engl J Med 365(3):213–221. 10.1056/NEJMoa1012592 PMC3153949 21774709

[10] Ankle Brachial Index Collaboration; Fowkes FG, Murray GD, Butcher I, Heald CL, Lee RJ, et al (2008) Ankle brachial index combined with Framingham Risk Score to predict cardiovascular events and mortality: a meta-analysis. JAMA 300(2):197–208. 10.1001/jama.300.2.197 PMC2932628 18612117

[11] Liang Y, Liu B, Xiao Y, Zeng X, Wu G, Du Z et al (2022) Retinal neurovascular changes in patients with ischemic stroke investigated by optical coherence tomography angiography. Front Aging Neurosci 14:834560. 10.3389/fnagi.2022.834560 PMC9289443 35860669

[12] Lim HB, Lee MW, Park JH, Kim K, Jo YJ, Kim JY (2019) Changes in ganglion cell-inner plexiform layer thickness and retinal microvasculature in hypertension: an optical coherence tomography angiography study. Am J Ophthalmol 199:167–176. 10.1016/j.ajo.2018.11.016 30502337

[13] Majithia S, Quek DQY, Chee ML, Lim ZW, Nusinovici S, Soh ZD et al (2023) Cardiovascular disease and thinning of retinal nerve fiber layer in a multi-ethnic Asian population: the Singapore epidemiology of eye diseases study. Front Med 10:1235309. 10.3389/fmed.2023.1235309 PMC10620687 37928469

[14] Neoh YL, Neoh PF, Salleh A, Yusof ZB, Gurusamy B, Ahmad Tajudin LS (2018) Triple vessel coronary artery disease and retinal nerve fibre layer thickness. Ann Acad Med Singap 47(6):226–229 30019068

[15] Chen Y, Yuan Y, Zhang S, Yang S, Zhang J, Guo X et al (2023) Retinal nerve fiber layer thinning as a novel fingerprint for cardiovascular events: results from the prospective cohorts in UK and China. BMC Med 21(1):24. 10.1186/s12916-023-02728-7 PMC9850527 36653845

[16] Cui X, Sun C, Wen D, Xiao J, Li X (2025) Causal effects between retinal characteristics and cardiovascular diseases: insights from genetic correlation, Mendelian randomization, and cross-sectional study. Glob Heart 20(1):104. 10.5334/gh.1493 PMC12636282 41282433

[17] Monteiro-Henriques I, Rocha-Sousa A, Barbosa-Breda J (2022) Optical coherence tomography angiography changes in cardiovascular systemic diseases and risk factors: a review. Acta Ophthalmol 100(1):e1–e15. 10.1111/aos.14851 33783129

[18] Zekavat SM, Sekimitsu S, Ye Y, Raghu V, Zhao H, Elze T et al (2022) Photoreceptor layer thinning is an early biomarker for age-related macular degeneration: epidemiologic and genetic evidence from UK Biobank OCT data. Ophthalmology. 129(6):694–707. 10.1016/j.ophtha.2022.02.001 PMC9134644 35149155

[19] Ritzel RM, Pan SJ, Verma R, Wizeman J, Crapser J, Patel AR et al (2016) Early retinal inflammatory biomarkers in the middle cerebral artery occlusion model of ischemic stroke. Mol Vis. 22:575–88 PMC4893077 27293375

[20] Kumar V (2017) Understanding retinal changes after stroke. Open J Ophthalmol. 7(4):281–292. 10.4236/ojoph.2017.74037 PMC6450536 30956896

[21] Sudlow C, Gallacher J, Allen N, Beral V, Burton P, Danesh J et al (2015) UK biobank: an open access resource for identifying the causes of a wide range of complex diseases of middle and old age. PLoS Med 12(3):e1001779. 10.1371/journal.pmed.1001779 PMC4380465 25826379

[22] Keane PA, Grossi CM, Foster PJ, Yang Q, Reisman CA, Chan K, UK Biobank Eye Vision Consortium et al (2016) Optical coherence tomography in the UK Biobank study - rapid automated analysis of retinal thickness for large population-based studies. PLoS ONE 11(10):e0164095. 10.1371/journal.pone.0164095 PMC5055325 27716837

[23] Wagner SK, Romero-Bascones D, Cortina-Borja M, Williamson DJ, Struyven RR, Zhou Y, UK Biobank Eye & Vision Consortium et al (2023) Retinal optical coherence tomography features associated with incident and prevalent Parkinson disease. Neurology 101(16):e1581–e1593. 10.1212/WNL.0000000000207727 PMC10585674 37604659

[24] Lau B, Cole SR, Gange SJ (2009) Competing risk regression models for epidemiologic data. Am J Epidemiol 170(2):244–256. 10.1093/aje/kwp107 PMC2732996 19494242

[25] R Core Team (2024). R: A language and environment for statistical computing. R Foundation for Statistical Computing, Vienna, Austria. URL http://www.R-project.org/. (2024, accessed 17 August 2026)

[26] Chen Y, Chen L, Li H, Yu Z (2026) The value of retinal microvascular features in predicting risk stratification and long-term prognosis of patients with acute coronary syndrome. BMC Ophthalmol 26(1):242. 10.1186/s12886-026-04769-x PMC13154654 41913145

[27] Rosinés-Fonoll J, Martin-Pinardel R, Marias-Perez S, Suarez-Valero X, Feu-Basilio S, Marín-Martinez S et al (2026) Optical coherence tomography angiography in type 1 diabetes mellitus. Report 5: cardiovascular risk. Biomedicines 14(1):153. 10.3390/biomedicines14010153 PMC12838605 41595687

[28] Cui J, Kwapong WR, Yan Y, Cao L, Ye C, Wang H et al (2024) Retinal sub-layer thicknesses, presence of lacunes, and their interaction with cognitive performance in recent single subcortical infarction. Chin Med J (Engl) 137(16):2014–2016. 10.1097/CM9.0000000000003224 PMC11332767 39033389

[29] Flammer J, Konieczka K, Bruno RM, Virdis A, Flammer AJ, Taddei S (2013) The eye and the heart. Eur Heart J 34(17):1270–1278. 10.1093/eurheartj/eht023 PMC3640200 23401492

[30] Muhammad IF, Borné Y, Zaigham S, Söderholm M, Johnson L, Persson M et al (2021) Comparison of risk factors for ischemic stroke and coronary events in a population-based cohort. BMC Cardiovasc Disord 21(1):536. 10.1186/s12872-021-02344-4 PMC8588679 34772344

[31] Campbell JP, Zhang M, Hwang TS, Bailey ST, Wilson DJ, Jia Y et al (2017) Detailed vascular anatomy of the human retina by projection-resolved optical coherence tomography angiography. Sci Rep 7:42201. 10.1038/srep42201 PMC5301488 28186181

[32] Chua J, Le TT, Sim YC, Chye HY, Tan B, Yao X et al (2022) Relationship of quantitative retinal capillary network and myocardial remodeling in systemic hypertension. J Am Heart Assoc. 11(6):e024226. 10.1161/JAHA.121.024226 PMC9075291 35253475

