# Supplementary material for: Retinal layer thickness as a potential marker for future cardiovascular events - a UK Biobank cohort study using optical coherence tomography
Source: J Neurol. 2026 Oct 15;273(11):662. doi: 10.1007/s00415-026-14175-4 (PMC13672466; doi:10.1007/s00415-026-14175-4)
Supplement: Supplementary file 1 [file 415_2026_14175_MOESM1_ESM.docx]

**Supplemental Online Content**

Hänsel M, Deseoe J, Herzog L, Davoudi N, Menze B, Sick B, Wegener S; Retinal layer thickness as a potential marker for future cardiovascular events – a UK Biobank cohort study using optical coherence tomography

**Additional methodical details and results**

**Supplementary Table S1.** Diagnosis codes used to identify cardiovascular risk factors

**Supplementary Table S2.** Cohort characteristics before and after exclusion of participants with insufficient OCT quality and missing covariates.

**Supplementary Table S3**. Linear regression model for association of cardiovascular risk factors with Retinal nerve fiber layer thickness in µm.

**Supplementary Table S4**. Linear regression model for association of cardiovascular risk factors with ganglion cell inner plexiform layer thickness in µm.

**Supplementary Table S5.** RNFL norm values (µm)

**Supplementary Table S6.** GCIPL norm values (µm)

**Supplementary Table S7.** Cause specific hazard models – unadjusted

**Supplementary Table S8**. All regression coefficients for fully adjusted cause specific hazard

model for association of GCIPL thickness with incident MACE

**Supplementary Table S9**. All regression coefficients for fully adjusted cause specific hazard

model for association of RNFL thickness with incident MACE

**Supplementary Table S10.** Subdistribution hazard models – unadjusted

**Supplementary Table S11.** Subdistribution hazard models - fully adjusted for CVRF

**Supplementary Table S12.** Cause specific hazard models - competing event (death)

**Supplementary Table S13.** Subdistribution hazard models - competing event (death)

**Supplementary Table S14.** Fully adjusted Cause specific hazard models - competing event (death)

**Supplementary Table S15.** Fully adjusted Subdistribution hazard models - competing event (death)

**Supplementary Table S16.** Evaluation of predictive performance - adding RNFL and GCIPL to a subdistribution hazard model based on CVRF

**Supplementary Figure S1**. Correlations of thicknesses of RNFL (left eye), RNFL (right eye), GCIPL (left eye) and GCIPL (right eye).

This supplementary material has been provided by the authors to give readers additional information about their work.

**Additional methodical details and results**

**1. Methods**

**Supplementary Table S1.** Diagnosis codes used to identify cardiovascular risk factors

| Disease | ICD-10 codes |
| --- | --- |
| Hypertension | I10 Essential (primary) Hypertension, I11 Hypertensive heart disease, I12 Hypertensive renal disease, I13 Hypertensive heart and renal disease, I15 Secondary hypertension |
| Diabetes | E10 Insulin dependent diabetes mellitus, E11 Non insulin dependent diabetes mellitus, E12 Malnutrition related diabetes mellitus, E13 Other specified diabetes mellitus, E14 unspecified diabetes mellitus |
| Atrial Fibrillation | G48 Atrial fibrillation and flutter |
| Dyslipidemia | E78 Disorders of lipoprotein metabolism and other lipidemias |

**Implementation**

All analyses were performed in the **R** programming language (version 4.4.0) using base packages and the following analysis-specific packages: survival [1] for survival analysis including cause specific and subdistribution hazard models, ggsurvfit for plotting cumulative incidence curves [2], ggplot2 for the other plots [3] and riskRegression for evaluation of model performance including calculation of ROC-AUC curves [4]

1. Therneau T (2024). *A Package for Survival Analysis in R*. R package version 3.8-3, [https://CRAN.R-project.org/package=survival](https://cran.r-project.org/package=survival).
2. Sjoberg D, Baillie M, Fruechtenicht C, Haesendonckx S, Treis T (2025). *ggsurvfit: Flexible Time-to-Event Figures*. R package version 1.1.0, <https://github.com/pharmaverse/ggsurvfit>.
3. Wickham H (2016). *ggplot2: Elegant Graphics for Data Analysis*. Springer-Verlag New York. ISBN 978-3-319-24277-4, [https://ggplot2.tidyverse.org](https://ggplot2.tidyverse.org/)
4. Gerds T, Ohlendorff J, Ozenne B (2025). *riskRegression: Risk Regression Models and Prediction Scores for Survival Analysis with Competing Risks*. [doi:10.32614/CRAN.package.riskRegression](https://doi.org/10.32614/CRAN.package.riskRegression), R package version 2025.05.20, [https://CRAN.R-project.org/package=riskRegression](https://cran.r-project.org/package=riskRegression).

**2. Results**

**Supplementary Table S2**. Cohort characteristics before and after exclusion of participants with insufficient OCT quality and missing covariates.

|  | Final cohort | Before Exclusion |
| --- | --- | --- |
| **n** | 51,293 | 59,469 |
| **Age (mean (SD))** | 56.83 (8.14) | 56.92 (8.14) |
| **Sex (Male) (%)** | 23,017 (44.9) | 26,863 (45.2) |
| **Hypertension (%)** | 12,775 (24.9) | 14,902 (25.1) |
| **Diabetes (%)** | 2,011 (3.9) | 2,414 (4.1) |
| **Dyslipidemia (%)** | 7,534 (14.7) | 87,89 (14.8) |
| **BMI [kg/m^2^]** | 27.25 (4.72) | 27.26 (4.73) |
| **Smoking (%)** |  |  |
| **Current** | 4,995 (9.7) | 5,758 (9.7) |
| **Never** | 28,580 (55.7) | 33,148 (55.9) |
| **Previous** | 17,718 (34.5) | 20,375 (34.4) |
| **Atrial fibrillation (%)** | 784 (1.5) | 921 (1.5) |

**Supplementary Table S3**. Linear regression model for association of cardiovascular risk factors with Retinal nerve fiber layer thickness in µm. P-values are shown for the corresponding linear regression coefficient in multivariate linear regression models.

| **RNFL** | **Estimate** | **Std. Error** | **P-value** |
| --- | --- | --- | --- |
| **(Intercept)** | 32.28 | 0.22 | < 0.001 |
| **Age [years]** | -0.04 | 0.00 | **< 0.001** |
| **Sex (Male)** | -1.05 | 0.06 | **< 0.001** |
| **BMI [kg/m^2^]** | -0.15 | 0.03 | **< 0.001** |
| **Hypertension** | -0.18 | 0.07 | **0.01** |
| **Diabetes** | -0.51 | 0.15 | **< 0.001** |
| **Dyslipidemia** | -0.13 | 0.09 | 0.14 |
| **Smoking (never)** | 0.37 | 0.10 | **< 0.001** |
| **Smoking (previous)** | 0.30 | 0.10 | **0.003** |
| **Atrial fibrillation** | -0.49 | 0.23 | **0.04** |

**Supplementary Table S4**. Linear regression model for association of cardiovascular risk factors with Ganglion cell inner plexiform layer thickness in µm. P-values are shown for the corresponding linear regression coefficient in multivariate linear regression models.

|  | **Estimate** | **Std. Error** | **P value** |
| --- | --- | --- | --- |
| **(Intercept)** | 80.46 | 0.30 | < 0.001 |
| **Age [years]** | -0.11 | 0.00 | **< 0.001** |
| **Sex (Male)** | 0.13 | 0.08 | 0.09 |
| **BMI [kg/m^2^]** | -0.03 | 0.04 | 0.50 |
| **Hypertension** | -0.59 | 0.10 | **< 0.001** |
| **Diabetes** | -0.61 | 0.20 | **0.003** |
| **Dyslipidemia** | -0.08 | 0.12 | 0.47 |
| **Smoking (never)** | -0.16 | 0.13 | 0.22 |
| **Smoking (previous)** | -0.13 | 0.14 | 0.37 |
| **Atrial fibrillation** | -1.15 | 0.32 | **< 0.001** |

**Supplementary Table S5.** RNFL thickness norm values (µm)

| **Age Group** | **Women (n)** | **Men**  **(n)** | **Mean RNFL thickness Women** | **Mean RNFL thickness Men** | **SD RNFL Women** | **SD RNFL Men** |
| --- | --- | --- | --- | --- | --- | --- |
| 40-44 | 2,613 | 2,183 | 30.82 | 29.63 | 5.10 | 4.77 |
| 45-49 | 3,794 | 2,882 | 30.77 | 29.63 | 5.10 | 4.56 |
| 50-54 | 4,439 | 3,178 | 30.11 | 29.33 | 5.07 | 4.71 |
| 55-59 | 4,970 | 3,541 | 30.10 | 28.96 | 5.16 | 4.85 |
| 60-64 | 6,469 | 5,205 | 29.91 | 28.90 | 5.23 | 4.87 |
| 65-69 | 4,500 | 4,201 | 29.60 | 28.59 | 5.57 | 5.12 |

**Supplementary Table S6.** GCIPL thickness norm values (µm)

| **Age Group** | **Women (n)** | **Men**  **(n)** | **Mean GCIPL thickness Women** | **Mean GCIPL thickness Men** | **SD GCIPL Women** | **SD GCIPL Men** |
| --- | --- | --- | --- | --- | --- | --- |
| 40-44 | 2,632 | 2,189 | 75.13 | 76.03 | 5.53 | 5.77 |
| 45-49 | 3,827 | 2,911 | 75.25 | 75.81 | 5.60 | 5.63 |
| 50-54 | 4,470 | 3,198 | 74.75 | 75.09 | 5.71 | 5.80 |
| 55-59 | 5,028 | 3,560 | 74.50 | 74.24 | 5.74 | 5.88 |
| 60-64 | 6,534 | 5,234 | 73.92 | 73.49 | 5.78 | 5.95 |
| 65-69 | 4,545 | 4,222 | 72.99 | 72.75 | 5.73 | 6.07 |

**Supplementary Table S7.** Cause specific hazard models – unadjusted. P values calculated for the Hazard ratio in unadjusted Cause specific hazard models.

| **MACE** | | | | |
| --- | --- | --- | --- | --- |
| **Layer** | **Hazard ratio** | **95% CI lower** | **95% CI upper** | **P - value** |
| GCIPL | 0.87 | 0.84 | 0.91 | < 0.001 |
| RNFL | 0.84 | 0.81 | 0.88 | < 0.001 |
| **Stroke** | | | | |
| **Layer** | **Hazard ratio** | **95% CI lower** | **95% CI upper** | **P - value** |
| GCIPL | 0.82 | 0.77 | 0.87 | < 0.001 |
| RNFL | 0.87 | 0.81 | 0.93 | < 0.001 |
| **Myocardial infarction** | | | | |
| **Layer** | **Hazard ratio** | **95% CI lower** | **95% CI upper** | **P - value** |
| GCIPL | 0.90 | 0.85 | 0.95 | < 0.001 |
| RNFL | 0.85 | 0.81 | 0.90 | < 0.001 |

**Supplementary Table S8.** All regression coefficients for fully adjusted cause specific hazard

model for association of GCIPL thickness with incident MACE. P values calculated using cause specific hazard models.

|  | **exp(coef)** | **se(coef)** | **Pr(>\|z\|)** |
| --- | --- | --- | --- |
| **Standardized GCIPL** | 0.96 | 0.02 | **0.038** |
| **Age [years]** | 1.08 | 0.003 | **< 0.001** |
| **Sex (male)** | 2.00 | 0.04 | **< 0.001** |
| **Hypertension** | 1.41 | 0.04 | **< 0.001** |
| **Diabetes** | 1.51 | 0.07 | **< 0.001** |
| **Dyslipidemia** | 1.02 | 0.05 | 0.717 |
| **BMI [kg/m^2^]** | 1.19 | 0.02 | **< 0.001** |
| **Atrial fibrillation** | 1.74 | 0.11 | **< 0.001** |
| **Smoking (Never)** | 0.45 | 0.06 | **< 0.001** |
| **Smoking (Previous)** | 0.43 | 0.06 | **< 0.001** |

**Supplementary Table S9.** All regression coefficients for fully adjusted cause specific hazard

model for association of RNFL thickness with incident MACE. P values calculated using cause specific hazard models.

|  | **exp(coef)** | **se(coef)** | **Pr(>\|z\|)** |
| --- | --- | --- | --- |
| **Standardized RNFL** | 0.92 | 0.02 | **< 0.001** |
| **Age [years]** | 1.08 | 0.003 | **< 0.001** |
| **Sex (male)** | 1.98 | 0.04 | **< 0.001** |
| **Hypertension** | 1.41 | 0.04 | **< 0.001** |
| **Diabetes** | 1.51 | 0.07 | **< 0.001** |
| **Dyslipidemia** | 1.02 | 0.05 | 0.721 |
| **BMI [kg/m^2^]** | 1.19 | 0.02 | **< 0.001** |
| **Atrial fibrillation** | 1.74 | 0.11 | **< 0.001** |
| **Smoking (Never)** | 0.46 | 0.06 | **< 0.001** |
| **Smoking (Previous)** | 0.43 | 0.06 | **< 0.001** |

**Supplementary Table S10.** Subdistribution hazard models – unadjusted. P values calculated for the Hazard ratio in unadjusted subdistribution hazard models.

| **MACE** | | | | |
| --- | --- | --- | --- | --- |
| **Layer** | **Hazard ratio** | **95% CI lower** | **95% CI upper** | **P - value** |
| GCIPL | 0.88 | 0.84 | 0.92 | < 0.001 |
| RNFL | 0.85 | 0.81 | 0.88 | < 0.001 |
| **Stroke** | | | | |
| **Layer** | **Hazard ratio** | **95% CI lower** | **95% CI upper** | **P - value** |
| GCIPL | 0.82 | 0.78 | 0.87 | < 0.001 |
| RNFL | 0.88 | 0.82 | 0.94 | < 0.001 |
| **Myocardial infarction** | | | | |
| **Layer** | **Hazard ratio** | **95% CI lower** | **95% CI upper** | **P - value** |
| GCIPL | 0.90 | 0.85 | 0.96 | < 0.001 |
| RNFL | 0.86 | 0.81 | 0.91 | < 0.001 |

**Supplementary Table S11.** Subdistribution hazard models - fully adjusted for CVRF. P values calculated for the Hazard ratio in adjusted subdistribution hazard models.

| **MACE** | | | | |
| --- | --- | --- | --- | --- |
| **Layer** | **Hazard ratio** | **95% CI lower** | **95% CI upper** | **P - value** |
| GCIPL | 0.96 | 0.91 | 1.01 | 0.08 |
| RNFL | 0.92 | 0.88 | 0.96 | < 0.001 |
| **Events: 2,526** | | **Median FU: 14.6 years** | | |
| **Stroke** | | | | |
| **Layer** | **Hazard ratio** | **95% CI lower** | **95% CI upper** | **P - value** |
| GCIPL | 0.90 | 0.84 | 0.97 | 0.004 |
| RNFL | 0.94 | 0.88 | 1.01 | 0.10 |
| **Events: 882** | | **Median FU: 14.7 years** | | |
| **Myocardial infarction** | | | | |
| **Layer** | **Hazard ratio** | **95% CI lower** | **95% CI upper** | **P - value** |
| GCIPL | 0.98 | 0.93 | 1.04 | 0.55 |
| RNFL | 0.94 | 0.89 | 1.00 | 0.04 |
| **Events: 1,411** | | **Median FU: 14.7 years** | | |

**Supplementary Table S12.** Cause specific hazard models - competing event (death). P values calculated for the Hazard ratio in unadjusted Cause specific hazard models.

| **MACE** | | | | |
| --- | --- | --- | --- | --- |
| **Layer** | **Hazard ratio** | **95% CI lower** | **95% CI upper** | **P - value** |
| GCIPL | 0.87 | 0.83 | 0.90 | < 0.001 |
| RNFL | 0.90 | 0.87 | 0.94 | < 0.001 |
| **Stroke** | | | | |
| **Layer** | **Hazard ratio** | **95% CI lower** | **95% CI upper** | **P - value** |
| GCIPL | 0.88 | 0.85 | 0.91 | < 0.001 |
| RNFL | 0.89 | 0.85 | 0.92 | < 0.001 |
| **Myocardial Infarction** | | | | |
| **Layer** | **Hazard ratio** | **95% CI lower** | **95% CI upper** | **P - value** |
| GCIPL | 0.86 | 0.83 | 0.89 | < 0.001 |
| RNFL | 0.88 | 0.85 | 0.91 | < 0.001 |

**Supplementary Table S13.** Subdistribution hazard models - competing event (death). P values calculated for the Hazard ratio in unadjusted subdistribution hazard models.

| **MACE** | | | | |
| --- | --- | --- | --- | --- |
| **Layer** | **Hazard ratio** | **95% CI lower** | **95% CI upper** | **P - value** |
| GCIPL | 0.87 | 0.84 | 0.91 | < 0.001 |
| RNFL | 0.91 | 0.87 | 0.95 | < 0.001 |
| **Stroke** | | | | |
| **Layer** | **Hazard ratio** | **95% CI lower** | **95% CI upper** | **P - value** |
| GCIPL | 0.88 | 0.85 | 0.92 | < 0.001 |
| RNFL | 0.89 | 0.85 | 0.92 | < 0.001 |
| **Myocardial infarction** | | | | |
| **Layer** | **Hazard ratio** | **95% CI lower** | **95% CI upper** | **P - value** |
| GCIPL | 0.86 | 0.83 | 0.92 | < 0.001 |
| RNFL | 0.88 | 0.85 | 0.91 | < 0.001 |

**Supplementary Table S14.** Fully adjusted Cause specific hazard models - competing event (death). P values calculated for the Hazard ratio in fully adjusted Cause specific hazard models.

| **Censored at MACE** | | | | |
| --- | --- | --- | --- | --- |
| **Layer** | **Hazard ratio** | **95% CI lower** | **95% CI upper** | **P - value** |
| GCIPL | 0.95 | 0.91 | 0.99 | 0.02 |
| RNFL | 0.97 | 0.93 | 1.01 | 0.09 |
| **Events: 2,628** | | |  |  |
| **Censored at Stroke** | | | | |
| **Layer** | **Hazard ratio** | **95% CI lower** | **95% CI upper** | **P - value** |
| GCIPL | 0.97 | 0.93 | 1.01 | 0.09 |
| RNFL | 0.96 | 0.92 | 0.99 | 0.02 |
| **Events: 3,231** | | |  |  |
| **Censored at Myocardial infarction** | | | | |
| **Layer** | **Hazard ratio** | **95% CI lower** | **95% CI upper** | **P - value** |
| GCIPL | 0.94 | 0.91 | 0.98 | 0.002 |
| RNFL | 0.94 | 0.91 | 0.98 | 0.002 |
| **Events: 3,235** | | |  |  |

**Supplementary Table S15.** Fully adjusted Subdistribution hazard models - competing event (death). P values calculated for the Hazard ratio in fully adjusted subdistribution hazard models.

| **Censored at MACE** | | | | |
| --- | --- | --- | --- | --- |
| **Layer** | **Hazard ratio** | **95% CI lower** | **95% CI upper** | **P - value** |
| GCIPL | 0.95 | 0.91 | 0.99 | 0.02 |
| RNFL | 0.97 | 0.93 | 1.01 | 0.09 |
| **Events: 2,628** | | |  |  |
| **Censored at Stroke** | | | | |
| **Layer** | **Hazard ratio** | **95% CI lower** | **95% CI upper** | **P - value** |
| GCIPL | 0.97 | 0.93 | 1.01 | 0.09 |
| RNFL | 0.96 | 0.92 | 0.99 | 0.02 |
| **Events: 3,231** | | |  |  |
| **Censored at Myocardial infarction** | | | | |
| **Layer** | **Hazard ratio** | **95% CI lower** | **95% CI upper** | **P - value** |
| GCIPL | 0.94 | 0.91 | 0.98 | 0.002 |
| RNFL | 0.94 | 0.91 | 0.98 | 0.002 |
| **Events: 3,235** | | |  |  |

**Supplementary Table S16.** Evaluation of predictive performance - adding RNFL and GCIPL to a subdistribution hazard model based on age and sex or age sex and CVRF. P-values calculated using DeLongs Test.

| **Model** | **AUC** | **95% CI** | **P - value** |
| --- | --- | --- | --- |
| Age + Sex | 0.7081 | 0.6999 – 0.7172 |  |
| Age + Sex + RNFL + GCIPL | 0.7102 | 0.7011 – 0.7178 | **0.045** |
| Age + Sex + CVRF | 0.7401 | 0.7321 - 0.7482 |  |
| Age + Sex + CVRF + RNFL + GCIPL | 0.7403 | 0.7323 - 0.7483 | **0.89** |

**Supplementary Figure S1**

Title: Correlations of thicknesses of RNFL (left eye), RNFL (right eye), GCIPL (left eye) and GCIPL (right eye).

Description: Density plots (diagonal) show distribution of measurements per eye and retinal layer stratified by sex. Left and right GCIPL thickness have a higher correlation compared to left and right RNFL in both sexes. Scatterplots show pairwise associations as indicated by axis labels on the top and on the right. Correlation coefficients are Pearson correlation coefficients. N = 51´293.
